# A new genus and species of fish parasitic cymothoid (Crustacea, Isopoda) from the Indian Ocean coast of South Africa, with a key to the externally attaching genera of Cymothoidae

**DOI:** 10.3897/zookeys.889.38638

**Published:** 2019-11-14

**Authors:** Niel L. Bruce, Rachel L. Welicky, Kerry A. Hadfield, Nico J. Smit

**Affiliations:** 1 Biodiversity & Geosciences Program, Queensland Museum, PO Box: 3300, South Brisbane BC, Queensland 4101, Australia; 2 Water Research Group, Unit for Environmental Sciences and Management, North-West University, Private Bag X6001, Potchefstroom, 2520, South Africa; 3 School of Aquatic and Fishery Sciences, University of Washington, 1122 NE Boat Street, Seattle, WA, 98105, USA

**Keywords:** coral reefs, external attaching parasites, Kwazulu-Natal, Pomacanthidae, Sodwana Bay, southern Africa

## Abstract

*Bambalocra intwala***gen. et sp. nov**. is described from Sodwana Bay, north-eastern South Africa. The monotypic genus is characterised by the broadly truncate anterior margin of the head with a ventral rostrum, coxae 2–5 being ventral in position not forming part of the body outline and not or barely visible in dorsal view, and the posterolateral margins of pereonites 6 and 7 are posteriorly produced and broadly rounded. The antennulae bases are widely separated, with both antennula and antenna slender. The species is known only from the type locality and the known hosts are species of Pomacanthidae (Angelfish). A revised key to the externally attaching genera of Cymothoidae is provided.

## Introduction

Taxonomic research on the family Cymothoidae Leach, 1814 has always been episodic (see Smit et al. 2014). In the latter part of the previous century Brusca (1981) revised the Cymothoidae of the East Pacific, Bruce (1986) revised the genus *Mothocya* Costa, in Hope 1851 and the Australian externally and gill-attaching genera (Bruce 1987a, b, c, 1990, 1991) while Williams and Bunkley-Williams (1978, 1980, 1981, 1994; Bunkley-Williams et al. 1998, 2006; Bunkley-Williams and Williams 1981, 1999, 2000) undertook major revisions of the Caribbean taxa as well as making a significant contribution to knowledge of the Japanese cymothoid fauna (Bunkley-Williams and Williams 1986; Williams and Bunkley-Williams 1986, 1994). A period of relative quietude followed until the revisionary work of Hadfield et al. (2010–2017) on the South African cymothoid fauna and the revision of Australian buccal attaching genera by Martin and Bruce (2014–2016).

In the period 1980 to the present day, despite the activity of the authors cited here, only six new cymothoid genera have been described (Boyko et al. 2019). The discovery, among specimens of unidentified Cymothoidae held at the South African Museum of a specimen that could not be placed into any existing externally attaching genus, is therefore of great interest. The species shares characters of *Renocila* Miers, 1880 and *Anilocra* Leach, 1818, but lacks the diagnostic characters of both genera. Equally the new species could not be placed into *Nerocila* Leach, 1818 or *Creniola* Bruce, 1987 (see generic ‘Remarks’). As the species does have a highly distinctive character, ventrally positioned and posteriorly acute coxal plates, we feel that the species is sufficiently distinctive to warrant a new genus.

The South African cymothoid fauna had remained little studied, with only 12 species in seven genera reported (Kensley 1978) until the recent revisions of Hadfield et al. (2010, 2013, 2014a, 2014b, 2015; Hadfield and Smit 2017), Van der Wal et al. (2017, 2019), and Welicky and Smit (2019), now with 21 species in eight genera. The major taxa remaining to be revised are the externally attaching genera *Nerocila* and *Renocila*, both of which have numerous unreported species in the Western Indian Ocean region (pers. obs.) and are currently under study. The diversity of cymothoids in this region is relatively high but, as is typical of the Cymothoidae, regional generic endemism is low with *Cinusa* Schioedte & Meinert, 1884 the only endemic genus (Hadfield et al. 2010). It is therefore of interest that a new and apparently endemic genus has been discovered in the subtropical Indian Ocean coast of South Africa.

## Materials and methods

Unidentified material from Sodwana Bay, South Africa, was loaned from the Iziko South African Museum (**SAMC**). Methods follow Hadfield et al. (2010) and van der Wal et al. (2019). Species descriptions were prepared using DELTA (Descriptive Language for Taxonomy), following a general Cymothoidae character data set originally developed by Hadfield et al. (2013) and recently updated (Hadfield et al. 2016). Fish nomenclature was taken from FishBase (Froese and Pauly 2019) and Catalog of Fishes (Eschmeyer 2019).

Abbreviations:

**RS** robust seta/e;

**SAMC** South African Museum, Cape Town;

**TL** total length;

**W** width.

## Taxonomy

### Suborder Cymothoida Wägele, 1989

#### Superfamily Cymothooidea Leach, 1814

**Family Cymothoidae Leach, 1814**

##### Genus Bambalocra gen. nov.

Taxon classificationAnimaliaIsopodaCymothoidae

AE819FCE-DE9D-5E90-847C-B2A5AE498B60

http://zoobank.org/F47D09E3-61CF-4A9F-B64C-2DD2D4C2796D

###### Type species.

*Bambalocra intwala* sp. nov.; original designation.

###### Etymology.

Bamba is an isiZulu word meaning ‘to grip’, combined with the ending –*locra*, alluding to related genera such as *Anilocra*; the name refers to the isopod gripping onto its host. Gender is female.

###### Diagnosis of female.

Cephalon anterior margin wide, with ventral posteriorly directed rostrum separating bases of antennulae; posterior margin not trilobed. Body weakly vaulted; coxae 2–6 ventral, not visible in dorsal view; posterolateral margins of pereonites 6 and 7 posteriorly produced, that of pereonite 7 extending posteriorly along pleon to pleonites 2 or 3. Pleonites all wide, posterolateral margins narrowly rounded to acute, pleonite 1 largely visible; pleonites 1–2 ventrolateral margins not produced. Antennula and antenna both slender, antenna longer than antennula; bases widely separate. Pleopods 1–2 lamellar, pleopods 3 and 4 endopod with small, weak lobes, pleopod 5 endopod with small fleshy lobes; peduncle of pleopods 2–5 with fleshy medial lobes present.

###### Additional characters.

Body twice as long as wide. Eyes posterolateral in position, less than 0.3 width of cephalon. Pereonite 2 shortest, 5 and 6 longest; pereonite 5 widest. Pleotelson flat. Mandible palp articles robust; article 3 broadly rounded, as long as proximal width. Maxillula with 4 terminal RS. Maxilla with 1 and 2 small recurved RS each on medial and lateral lobe, respectively. Maxilliped articles broad, article 3 with 3 RS. Pereopods 1–5 subequal in length, 6 slightly longer than 1–4; pereopod 7 longer than pereopod 6. Brood pouch formed by two large oostegites arising from pereonite 6, smaller alternately overlapping oostegites arising from pereonites 1–4, posterior pocket present. Uropod rami subequal in length, visible in dorsal view, slightly exceeding pleotelson posterior margin.

###### Male

**(juvenile)**. Smaller, narrower, less ovate than female; pereonites 2–7 posterolateral angles rounded, not or weakly posteriorly produced. Appendages similar to female, except folds on pleopods 3–5 endopod absent.

###### Remarks.

*Bambalocra* gen. nov. can immediately be identified and distinguished from all other marine cymothoid genera by coxae 2–5 being ventral in position, not forming part of the body outline and not or barely visible in dorsal view, all are posteriorly acute, and the posterolateral margins of pereonite 6 and 7 are posteriorly produced and broadly rounded. The antennula bases are widely separated, with both antennula and antenna slender.

*Bambalocra* gen. nov. superficially resembles *Renocila* in having a relatively broad body with a weakly vaulted dorsum and the posterolateral margins of pereonites 6 and 7 expanded and posteriorly directed; in dorsal view the anterior margin of the cephalon appears similar, being weakly produced and wide. Unlike *Renocila* the ventral rostrum is triangular (vs. broadly truncate in *Renocila)* and is posteriorly directed separating the antennular bases (vs. not posteriorly directed between the antennular bases); pleonite 1 in *Bambalocra* is not markedly narrower than the remaining pleonites (vs. narrower, which is diagnostic for *Renocila*). Most species of *Renocila* have the antennula both longer and larger than the antenna, usually with strongly flattened expanded articles, while in *Bambalocra* the antennula is shorter than the antenna and both are slender; in most species of *Renocila* the coxae of pereonites 2–4 or 2–5 are visible in dorsal view.

Both *Nerocila* and *Creniola* have the posterior margin of the cephalon strongly trilobed, contrasting strongly to that of *Bambalocra*. Species of *Nerocila* are characterised by having pleonites 1 and 2 with ventral processes, while in *Creniola* the pleon is as wide or wider than the pereon. In both these genera the coxae are conspicuous in dorsal view. Most species of *Anilocra* have a relatively elongate body, with a strongly vaulted dorsum; the coxae and the posterolateral margins of pereonites 5–7 are neither expanded nor posteriorly produced.

##### Bambalocra intwala sp. nov.

Taxon classificationAnimaliaIsopodaCymothoidae

707B590A-680E-56C8-A4B7-EC8E4736F6B9

http://zoobank.org/6C41B5E7-9853-48E9-8DBF-3BDD1661AE07

**Figure 1.**
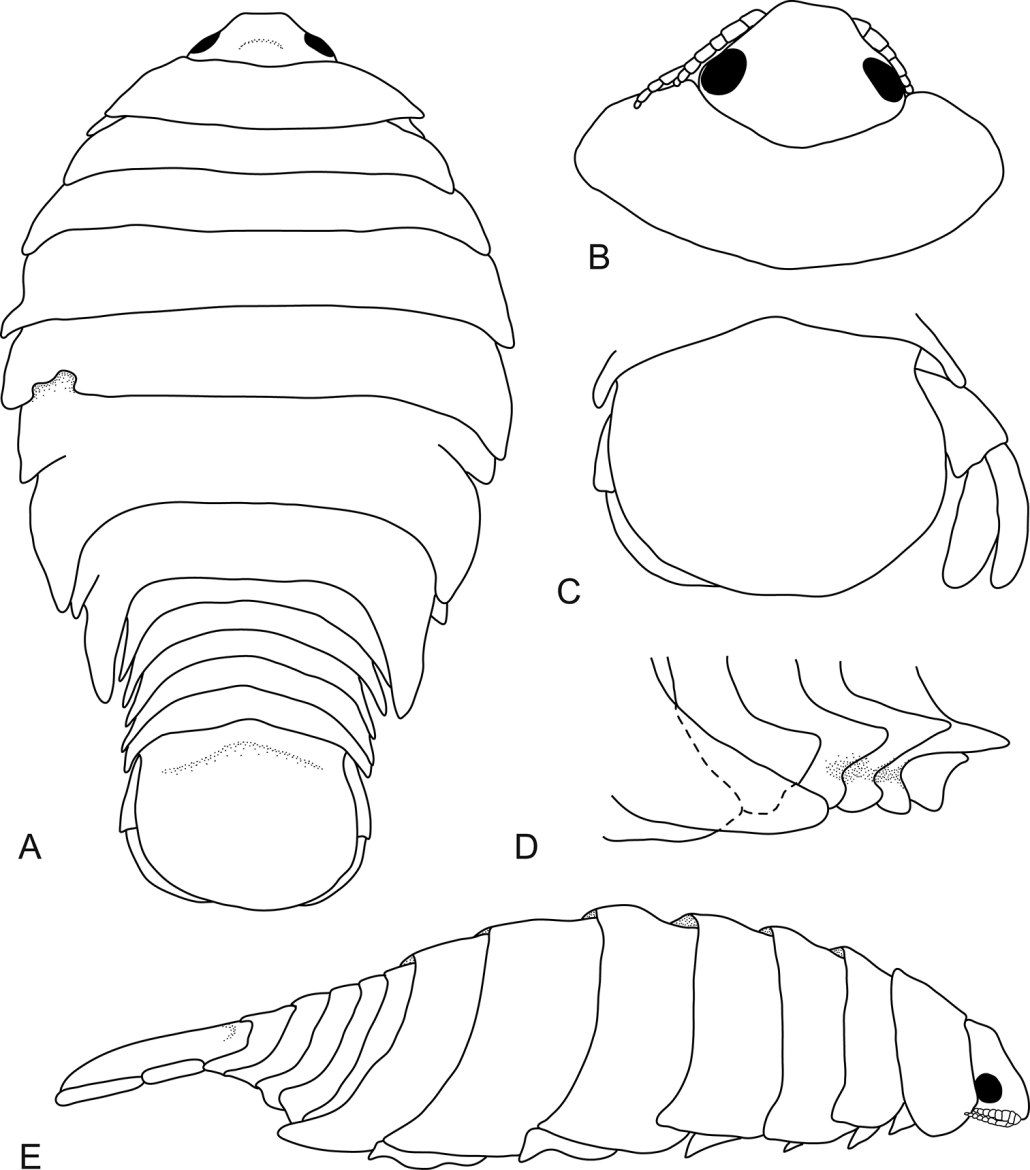
*Bambalocra intwala* sp. nov. Holotype, 23.5 mm (SAMC-A091364) **A** dorsal view **B** dorsal view, head, pereonite 1 **C** pleotelson **D** pleonites, lateral view **E** lateral view.

**Figure 2.**
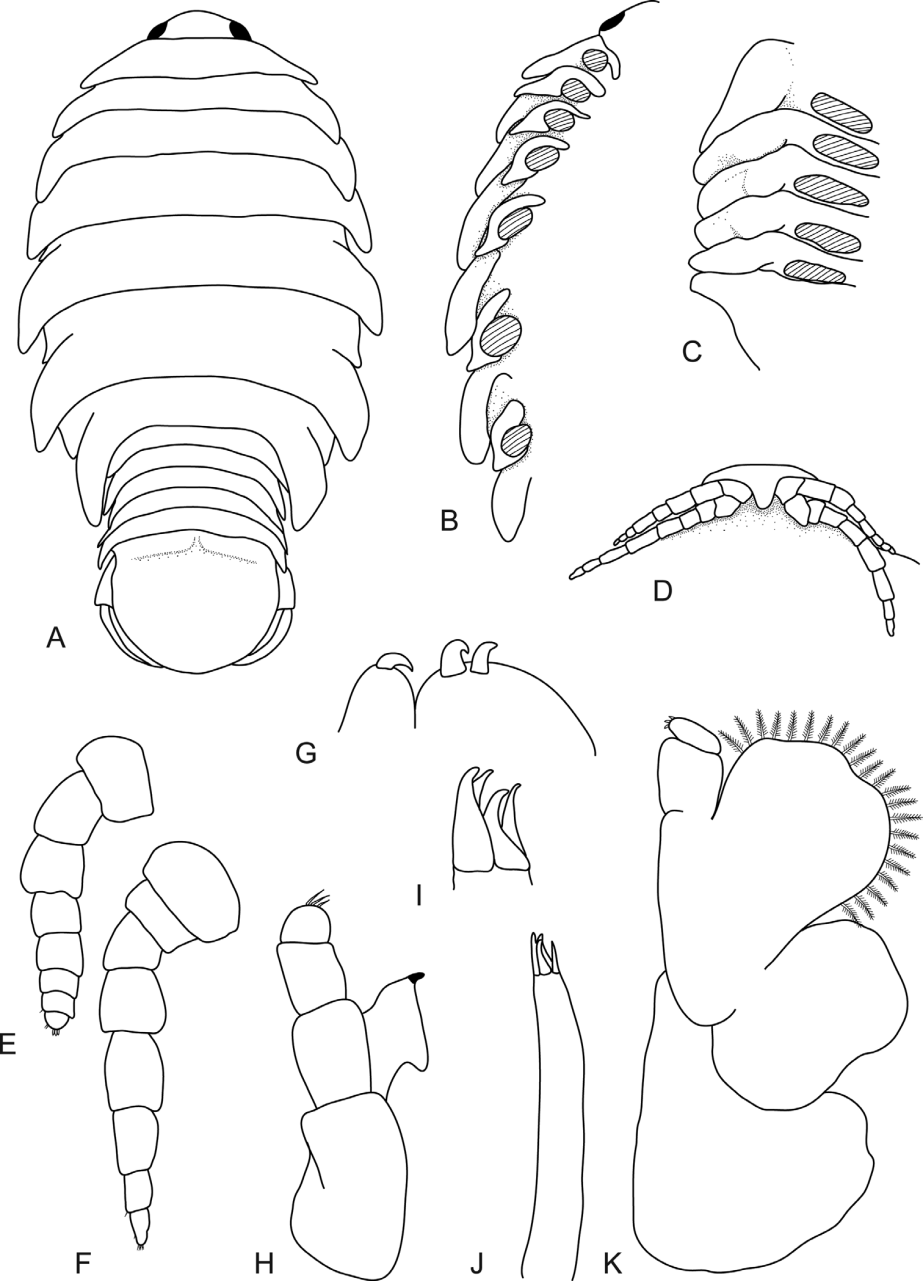
*Bambalocra intwala* sp. nov. Female paratype, 23.0 mm (SAMC-A091365) **A** dorsal view **B** coxae, ventral view **C** pleonites, ventral view (pleopods removed) **D** frons **E** antennula **F** antenna **G** maxilla apex **H** mandible **I** maxillula apex **J** maxillula **K** maxilliped.

**Figure 3.**
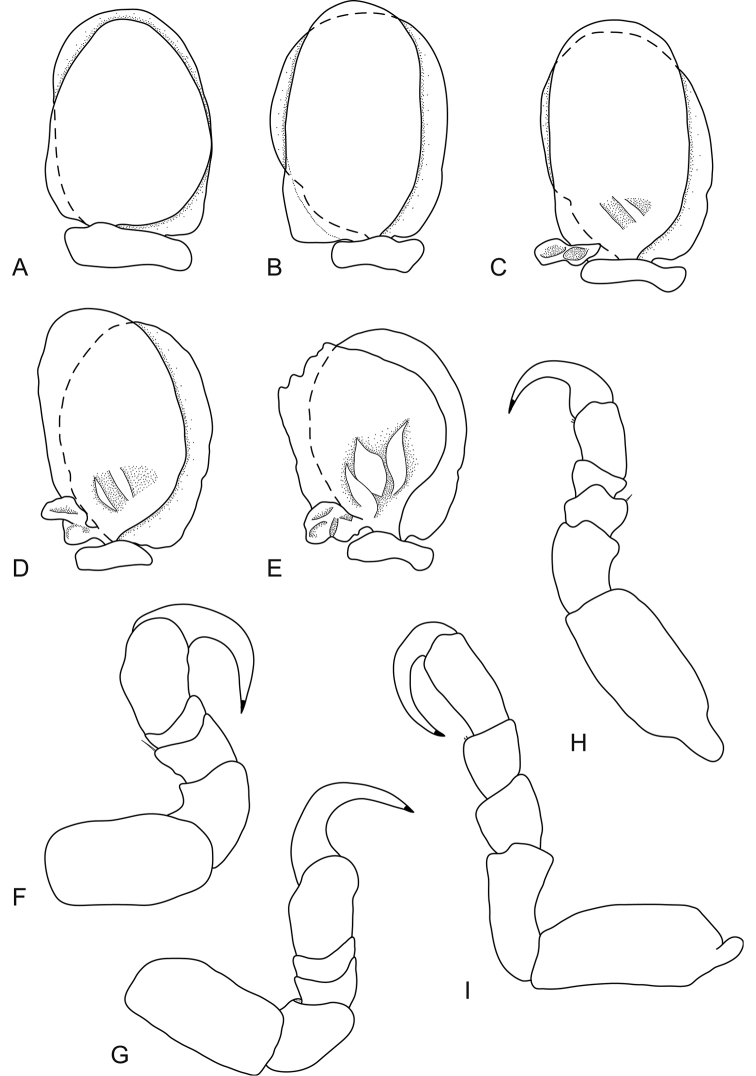
*Bambalocra intwala* sp. nov. Female paratype, 23.0 mm (SAMC-A091365) **A–E** pleopods 1–5 respectively **F** pereopod 1 **G** pereopod 2 **H** pereopod 6 **I** pereopod 7.

**Figure 4.**
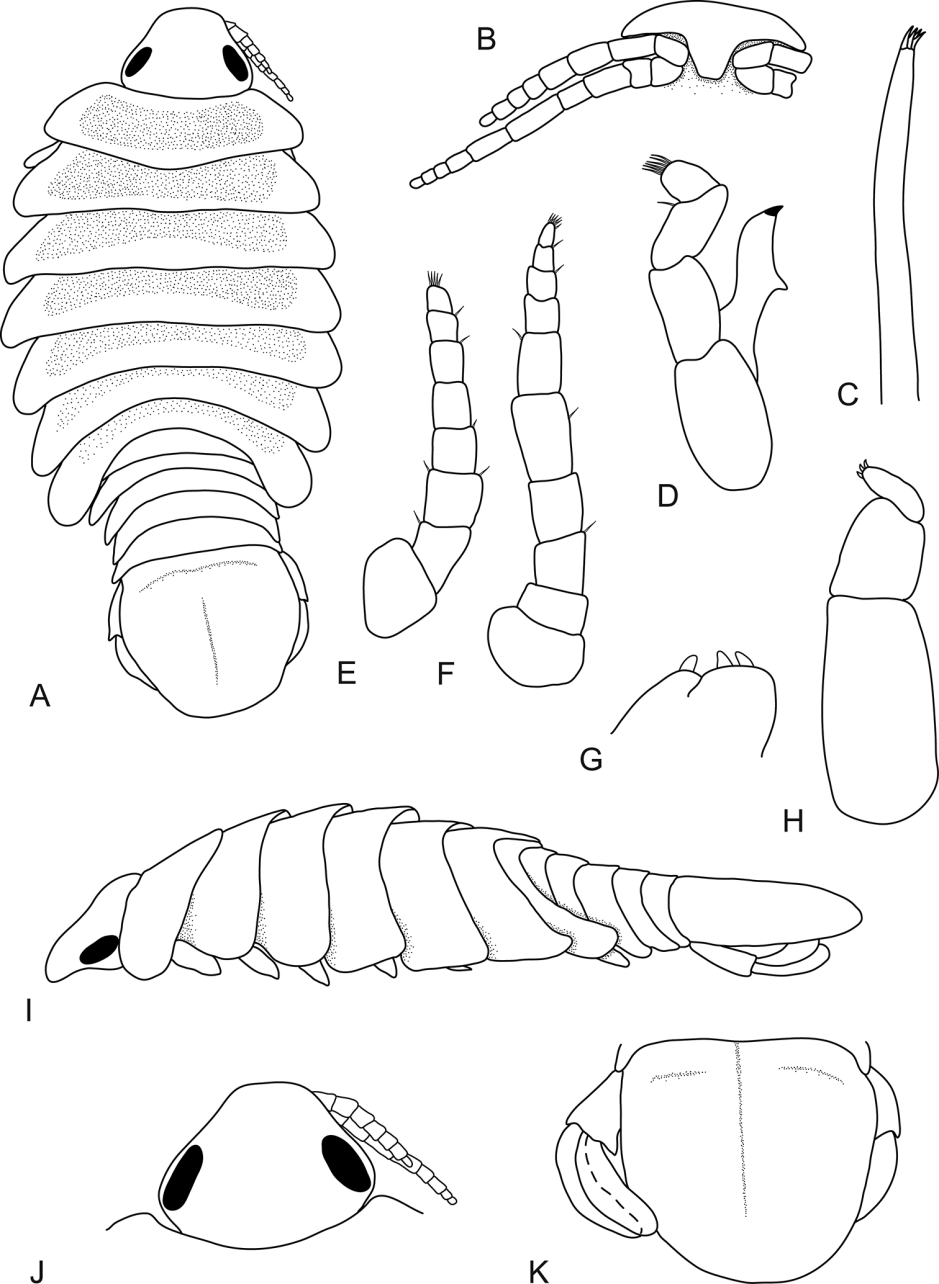
*Bambalocra intwala* sp. nov. Male paratype, 7.5 mm (SAMC-A091365) **A** dorsal view **B** frons **C** maxillula **D** mandible **E** antennula **F** antenna **G** maxilla apex **H** maxilliped **I** lateral view **J** head, dorsal view **K** pleotelson and uropods.

**Figure 5.**
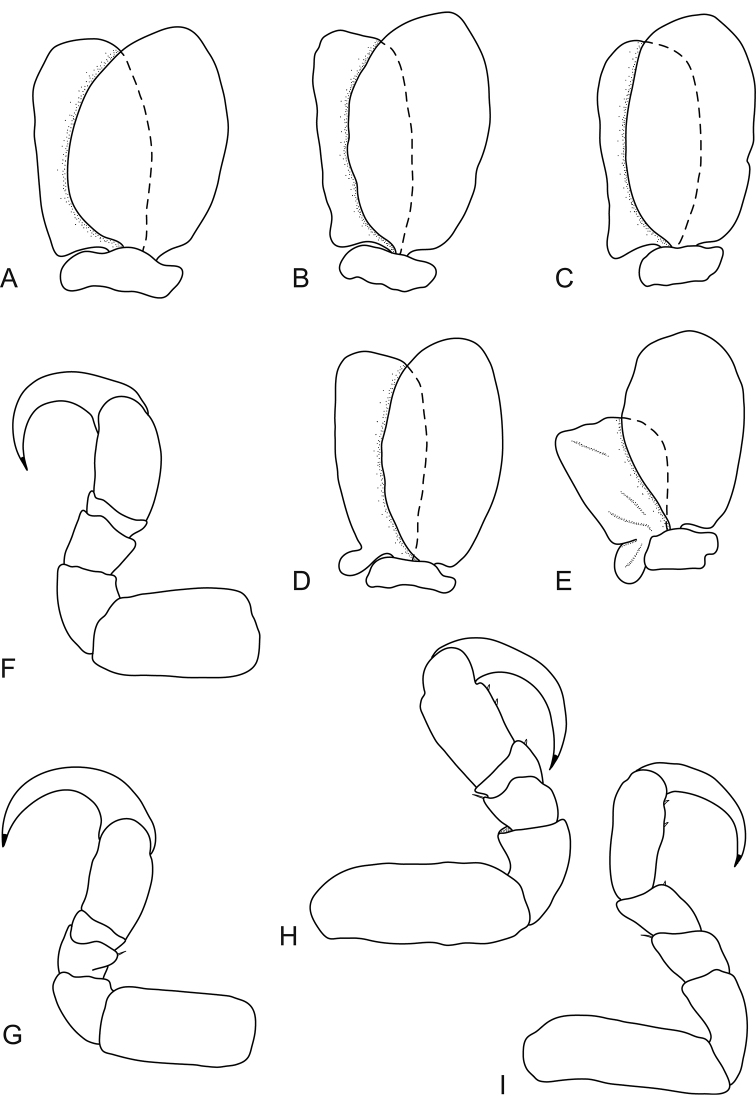
*Bambalocra intwala* sp. nov. Male paratype, 7.5 mm (SAMC-A091365) **A–E** pleopods 1–5 respectively **F** pereopod 1 **G** pereopod 2 **H** pereopod 6 **I** pereopod 7.

Figures 1 , 2 , 3 , 4 , 5

###### Material examined.

***Holotype***: South africa • 1 ♀ (ovigerous, 23.5 mm TL, 12 mm W); Sodwana Bay, Kwazulu-Natal; 27°32'S, 32°41'E; April 1979; host not recorded, coll. R.E. Stobbs; SAMC-A091364.

***Paratypes***: South africa • 1 ♂ (immature, 7.5 mm TL, 3.0 mm W) 3 ♀♀ (23–24 mm TL, 11.0–12.0 mm W); same data as holotype; SAMC-A091365 • 1 ♀ (20.0 mm TL, 11.0 mm W); Sodwana Bay, Kwazulu-Natal; 27°30'S, 32°41'E; 12.8 m depth; July 1976; host not recorded, coll. Richard Winterbottom (RW 76-14); SAMC-A091366 • 1 ♀ (21.0 mm TL, 11.5 mm W); Durban Sea World; September 2003; from a dwarf angelfish (*Centropyge*); SAMC-A091367.

###### Description of female

(from holotype and 23.0 mm female paratype). *Body* oval, 1.9 times as long as greatest width, dorsal surfaces smooth, widest at pereonite 5, narrowest at pereonite 1; lateral margins ovate. *Cephalon* 0.7 times longer than wide, *frontal margin* broadly truncate. *Eyes* oval with distinct margins, eye width 0.2 times width of cephalon. Pereonite 1 anterior border anteriorly concave, anterolateral angles narrowly rounded. Coxae 2 and 3 narrow with posteroventral angles with small distinct produced point; coxae 4–7 with small, distinct dorsally directed point, not extending past pereonite margin. Pereonites 4–7 with posteroventral angle weakly produced, acute; pereonite 7 posterolateral margins extending to pleonite 3. *Pleon* 0.4 times as wide as pereon. Pleonites posterior margin evenly concave; pleonite 1 widest, visible in dorsal view; pleonite 2 partially overlapped by pereonite 7; posterolateral angles of pleonite 2 narrowly rounded. Pleonites 3–5 similar in form to pleonite 2; pleonite 5 not overlapped by lateral margins of pleonite 4, posterior margin slightly concave. *Pleotelson* 0.8 times as long as anterior width, dorsal surface smooth, lateral margins convex, posterior margin evenly rounded.

*Antennula* length shorter than antenna, extending to anterior of pereonite 1, consisting of 8 articles; peduncle articles 1 and 2 distinct and articulated; article 2 1.7 times as long as article 1; 0.4 times as long as combined lengths of articles 1 and 2. *Antenna* extending to anterior margin of pereonite 1, consisting of 8 articles; article 3 1.4 times as long as article 2; article 4 1.4 times as long as article 3; article 5 1.4 times as long as article 4; terminal article with 3 short simple setae distally.

*Mandibular molar process* present, small; palp article 3 with 3 simple setae. *Maxillula* with lateral RS largest. *Maxilliped palp* consisting of 3 articles, with lamellar oostegite lobe; article 2 without setae, article 3 with 3 recurved short RS.

*Pereopod 1* basis 1.7 times as long as greatest width; ischium 0.5 times as long as basis; merus proximal margin without bulbous protrusion; propodus 1.5 times as long as wide; dactylus moderately slender, 1.3 times as long as propodus, 3.2 times as long as basal width. *Pereopod 2* propodus 1.4 times as long as wide; dactylus 1.5 times as long as propodus. *Pereopod* 3 similar to pereopod 2. *Pereopod 6* basis 2.1 times as long as greatest width, ischium 0.3 times as long as basis; propodus 1.2 times as long as wide; dactylus 2.1 times as long as propodus. *Pereopod 7* longer than other pereopods, slightly longer than pereopod 6; basis 2.3 times as long as greatest width; ischium 0.7 times as long as basis, without protrusions; merus proximal margin without bulbous protrusion, as long as wide, 0.4 times as long as ischium; carpus 1.2 times as long as wide, 1.1 times as long as ischium, without bulbous protrusion; propodus 1.9 times as long as wide, 1.4 times as long as ischium; dactylus moderately slender, 1.6 times as long as propodus, 3.5 times as long as basal width.

*Pleopods* 1 and 2 rami simple, 3–5 endopods with weak fleshy ridges. *Pleopod 1* exopod 1.4 times as long as wide, lateral margin weakly convex, distally broadly rounded, mesial margin weakly convex; endopod 1.2 times as long as wide, lateral margin convex, distally broadly rounded, mesial margin slightly convex; peduncle 2.8 times as wide as long. Pleopod endopods 3–5 each with proximomedial lobe.

*Uropod* 0.8 times as long as pleotelson; peduncle 0.7 times as long as rami, lateral margin without setae; rami not extending beyond pleotelson, marginal setae absent, apices broadly rounded. *Endopod* 2.9 times as long as greatest width, as long as exopod, lateral margin convex, mesial margin straight. *Exopod* extending to end of endopod, 3.1 times as long as greatest width, lateral margin convex.

###### Male

(juvenile paratype 7.5 mm) . Body approximately 2.0 times longer than wide; posterolateral margins of pereonites laterally extending giving indented body outline; coxae posteriorly acute; pereonite 7 extending to posterior of pleonite 2. Pleon half as wide as body, all pleonites visible in dorsal view. *Pleotelson* 0.9 times as long as anterior width, lateral margins convex, posterior margin evenly rounded.

*Antennula* with 8 articles. Antenna with 10 articles. Mandible article 3 with 6 RS. Pereopods similar in proportions to female. Pleopods similar in proportions to female; pleopod 2 appendix masculina and penial processes absent.

*Uropod* 0.8 as long as pleotelson, peduncle 0.5 times as long as rami, rami not extending beyond pleotelson, apices narrowly rounded. *Endopod* 4.0 times as long as greatest width, 0.8 as long as exopod, lateral margin weakly convex, mesial margin weakly concave. *Exopod* extending beyond end of endopod, 4.7 times as long as greatest width, lateral margin convex, mesial margin concave.

###### Remarks.

As the genus is monotypic, the species is identified by the generic characters, in particular the coxae being ventral in position rather than lateral, the pleonites being all wide, without ventrolateral processes, in combination with the short posteriorly directed ventral rostrum that separates the slender antennula and antenna.

###### Host.

There is no host data for the holotype and wild-caught paratypes; the specimen from the Durban Aquarium is from a dwarf angelfish (*Centropyge*). Several species of externally attaching cymothoid have been photographed by SCUBA divers on the coral reefs at Sodwana Bay; from the photographs three species of Pomacanthidae (angelfish) are identified as probable hosts: *Apolemichthys trimaculatus* (Cuvier, 1831), *Pomacanthus imperator* (Bloch, 1787), and *Pygoplites diacanthus* (Boddaert, 1772), but these hosts need to be confirmed by direct capture of the isopods in situ.

###### Etymology.

The epithet is the word for louse (*intwala*) in the isiZulu language (noun in apposition).

#### Key to the externally attaching genera of the Cymothoidae (modified after Bruce, 1987)

**Table:** 

1	Cephalon posterior margin trisinuate; coxae 5–7 as long as or longer than respective pereonite (except *N. lomatia*)	**2**
–	Cephalon posterior margin not trisinuate; coxae 5–7 manifestly shorter than respective pereonite	**5**
2	Pleonites 1 and 2 ventrolateral margins produced	*** Nerocila ***
–	Pleonites 1 and 2 ventrolateral margins not produced	**3**
3	Body dorsal surface strongly vaulted; coxae 5–7 ventrally directed; pleonites strongly produced ventrally	*** Plotor ***
–	Body dorsal surface weakly vaulted; coxae 5–7 posteriorly directed; pleonites not strongly produce ventrally	**4**
4	Uropod rami long, extending well beyond posterior of pleotelson; coxae conspicuous in dorsal view	*** Amblycephalon ***
–	Uropod rami short, not extending beyond posterior of pleotelson; coxae inconspicuous in dorsal view	*** Creniola ***
5	Cephalon without rostrum, or rostrum not projecting between antennula bases; antennula broader than and as long as, or longer than antenna; posterolateral margins of pereonites 5–7 produced; coxae 5–7 posteriorly acute	*** Renocila ***
–	Rostrum folded under, projecting between antennula bases; antennula more slender than and shorter than antenna; posterolateral margins of pereonites 6 and 7 produced (*Bambalocra*) or not produced coxae posteriorly rounded	**6**
6	Coxae ventral in position, not or barely visible in dorsal view; posterolateral margins of pereonites 6 and 7 posteriorly produced, rounded; pleopods 1 and 2 lamellar, pleopods 3–5 endopods with weak lobes; pleopods hardly visible in dorsal view	***Bambalocra* gen. nov.**
–	Coxae lateral in position, largely not visible in dorsal view, posterolateral margins of pereonites 5–7 not produced; pleopod 5 with prominent folded fleshy lobes; pleopods clearly visible in dorsal view	**7**
7	Mandible palp article 3 shorter than article 2; maxilla with 2 short hooked RS each on medial and lateral lobe, medial lobe partly fused to lateral; antennula articles 4–8 short; pleonites 3–5 or 4 and 5 more than half width (ca. 0.70) of pereon	*** Anilocra ***
–	Mandible palp article 3 longer than article 2; maxilla with 2 large nodular RS each on medial and lateral lobe, medial lobe distinct; antennula articles 4–8 elongate; pleonites 3–5 or 4 and 5 less than half width (ca. 0.45) of pereon	*** Pleopodias ***

## Supplementary Material

XML Treatment for Bambalocra

XML Treatment for Bambalocra intwala
